# Novel histone acetylation-related lncRNA signature for predicting prognosis and tumor microenvironment in esophageal carcinoma

**DOI:** 10.18632/aging.205636

**Published:** 2024-03-13

**Authors:** Batter Han, Ying Ma, Pengjie Yang, Fangchao Zhao, Haiyong Zhu, Shujun Li, Rong Yu, Subudao Bao

**Affiliations:** 1 Department of Thoracic Surgery, Peking University Cancer Hospital Inner Mongolia Hospital, Hohhot 010010, China; 2 Department of Thoracic Surgery, Mongolia Medical University Affiliated Hospital, Hohhot 010050, China; 3 Department of Thoracic Surgery, The Second Hospital of Hebei Medical University, Shijiazhuang 050000, China; 4 Mongolian Medicine College, Inner Mongolia Medical University, Hohhot 010110, China

**Keywords:** histone acetylation, esophageal carcinoma, tumor microenvironment, immunotherapy

## Abstract

Histone acetylation is one of the most common epigenetic modifications and plays a crucial role in tumorigenesis. However, the prognostic significance of histone acetylation-related lncRNAs (HARlncRNAs) in esophageal carcinoma (ESCA) is not well understood. A total of 653 differentially expressed lncRNAs (DElncRNAs) were identified between 162 ESCA tissues and 11 normal tissues in the TCGA database, and 7 of them were correlated with acetylation regulators. We employed univariate Cox regression analysis, combining it with clinical prognosis information, to select 3 prognostic-related HARlncRNAs for further analysis. Subsequently, we used LASSO regression analysis to construct a risk signature for ESCA and identified *C21orf62-AS1* and *SSTR5.AS1* as potential biomarkers for the prognosis of ESCA patients. Based on the risk score calculated using the risk signature, we categorized patients into high- and low-risk groups. We identified the risk score as an independent risk factor and validated it in the training, test, and GSE53624 datasets. Additionally, patients categorized by their risk scores exhibited distinct immune statuses, tumor mutation burdens, responses to immunotherapy, and drug sensitivities.

## INTRODUCTION

Esophageal cancer (ESCA) ranks eighth in global cancer morbidity and sixth in cancer mortality, making it a significant threat to human life and health [1]. In China, esophageal squamous cell carcinoma (ESCC) constitutes over 90% of esophageal cancer cases, making it the predominant histological type [2]. Surgery-based comprehensive treatment is the primary approach for early and mid-stage ESCA, yielding a 5-year survival rate of 30–50%. Notably, postoperative local recurrence and distant metastasis are two significant contributing factors to this rate [3]. Recently, targeted therapy and emerging immunotherapy have created new avenues for ESCA treatment [4, 5]. Nonetheless, further investigation is required to explore new molecular-level therapeutic targets and prognostic biomarkers for ESCA.

Epigenetic modifications are genetic modifications that cause heritable phenotypic changes in the expression of a gene without altering its DNA sequence, which mainly include DNA methylation, histone modifications and non-coding RNAs [6, 7]. Acetylation, one of the common histone modifications, can affect protein function through a variety of mechanisms, including regulation of protein stability, enzymatic activity, subcellular localization, interaction with other post-translational modifications, and control of protein-protein DNA interactions, which in turn are involved in almost all important biological processes such as chromatin remodeling, transcription factor activation and metabolic regulation [8, 9]. However, imbalances in histone acetylation can lead to abnormal gene expression and alterations in important physiological functions, leading to disease onset or progression and tumor formation [10]. Emerging studies have shown that histone acetylation is involved in the development of various cancers by regulating various biological processes, including proliferation [11], apoptosis [12], metastasis [13], stemness [14], and drug sensitivity [15].

Long non-coding RNAs (lncRNAs) are RNA molecules longer than 200 nucleotides that lack the ability to code for proteins [16]. Over the last twenty years, lncRNAs have become pivotal in governing gene expression in various biological processes, including transcriptional regulation and epigenetic modifications. They participate in epigenetic processes by recruiting histone-modifying enzymes and DNA methyltransferases. This leads to the creation of chromatin conformation patterns, ultimately enabling precise gene regulation [17–19]. Some of these lncRNAs are related to tumorigenesis. In particular, histone acetylation-related lncRNAs (HARlncRNAs) play a crucial role in tumorigenesis and development, such as *SNHG14* in hepatocellular carcinoma [20], *MIR22HG* in liver cancer [21], *EIF3J-AS1* in colorectal cancer [22], and lncRNA *JADE* in breast tumorigenesis [23]. However, the relationship between HARlncRNAs and ESCA prognosis and tumor microenvironment (TME) has not been evaluated so far.

In our current study, we, for the first time, explored the role of HARlncRNAs in ESCA. We presented a risk signature model that uses genes linked to histone acetylation for predicting the prognosis of ESCA patients. We identified two histone acetylation-related genes (HARGs) for building the risk signature. This signature was confirmed as an independent risk factor and validated across the training, testing, and validation sets. Additionally, we conducted an in-depth analysis of the tumor microenvironmental characteristics in distinct HARlncRNA subgroups and assessed their responses to adjuvant therapy and immunotherapy. Our conclusion is that the HARlncRNA signature serves as a robust prognostic biomarker, capable of accurately predicting the response to adjuvant therapy and immunotherapy in ESCA.

## RESULTS

### Genetic variation landscape of HARGs in ESCA

**Figure 1:**
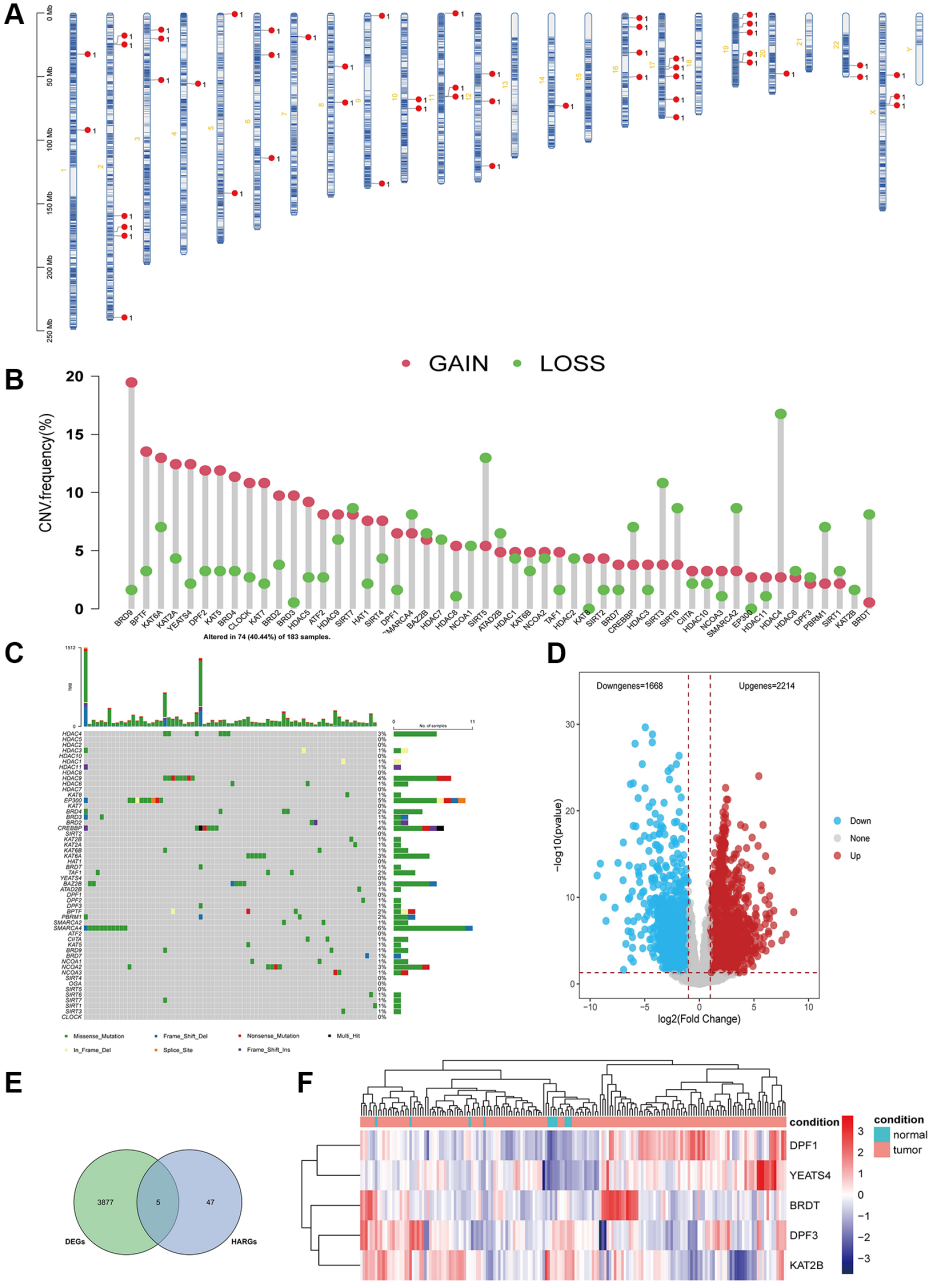
**Genetic mutational landscape of HARGs in ESCA.** (**A**) Chromosome distributions of HARGs. (**B**) The CNV mutation frequency of HARGs. (**C**) Somatic mutation spectrums of HARGs. (**D**) The volcano plot displayed down-regulated and up-regulated HARGs. (**E**) Venn diagram to identify 5 overlapping differentially expressed HARGs. (**F**) Heatmap shows 5 HARGs expression profiles among normal and ESCA samples.

In this study, we included 52 HARGs (Supplementary Table 1), and their positions on the chromosomes are displayed in Figure 1A. HARGs exhibited extensive CNV alterations in ESCA, with the majority of them involving copy number amplification (Figure 1B). As shown in Figure 1C, 74 out of 183 (40.44%) ESCC samples displayed genetic mutations, with *SMARCA4* having the highest mutation frequency. We assessed whether the genetic variation mentioned earlier influences mRNA expression in ESCA by comparing gene expression levels in both normal and tumor tissues, set a threshold of |Log2 Fold Change|>1 and *P*-value < 0.05 to screen for the differentially expressed genes (DEGs) (Figure 1D), and identified five differentially expressed HARGs by intersecting the DEGs and HARGs, as illustrated in Figure 1E. The expression of HARGs with higher amplification frequency was significantly elevated in tumor tissues compared to normal tissues, and the reverse was also true (Figure 1F). These findings imply that CNV may underlie the differences in HARGs expression. Additionally, the expression of HARGs exhibited significant heterogeneity in both normal and tumor tissues. This indicates that differential HARGs expression plays a crucial role in the onset and progression of ESCA.

### Identification of HARlncRNAs

**Figure 2:**
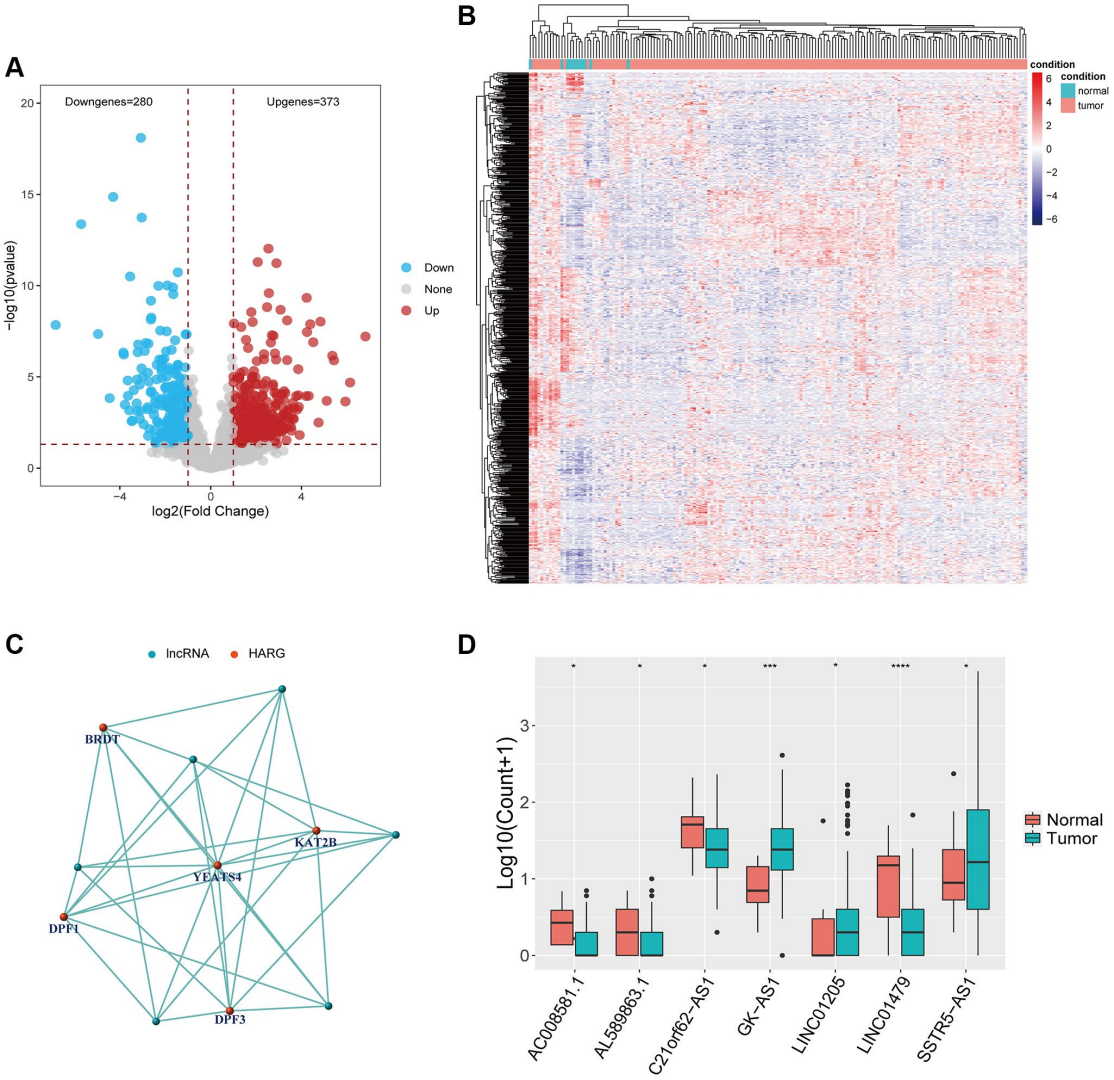
**Identification of HARlncRNAs in ESCA.** (**A**) Volcano plot of differentially expressed lncRNAs between tumor tissues and normal tissues. (**B**) Heatmap of differentially expressed lncRNAs between tumor tissues and normal tissues. (**C**) Co-expression relationship between HARlncRNAs and HARGs. (**D**) Scatter diagram indicated the different expression of HARlncRNAs in normal and tumor tissues. ^*^P < 0.05; ^**^P < 0.01; ^***^P < 0.001.

The identification of HARlncRNAs in ESCA relied primarily on correlation network analysis. Initially, we screened for DElncRNAs in both normal and tumor tissues using the same threshold, resulting in 373 up-regulated lncRNAs and 280 down-regulated lncRNAs (Figure 2A). Figure 2B displayed the expression patterns of DElncRNAs in normal tissues compared to tumor tissues. Using the expression values of HARGs and lncRNAs from 173 ESCA samples, we identified HARlncRNAs through the Pearson correlation analysis algorithm. We set a filter criterion of correlation coefficient >0.4 and *P*-value < 0.001. We constructed a correlation network using the final set of 7 screened HARlncRNAs to show their co-expression relationships (Figure 2C). Out of the seven HARlncRNAs, *GK-AS1*, *LINC01205*, and *SSTR5-AS1* exhibited high expression in tumor tissues, while *AC008581.1*, *AL589863.1*, *C21orf62-AS1*, and *LINC01479* displayed low expression in tumor tissues (Figure 2D).

### Construction of a risk signature

**Figure 3:**
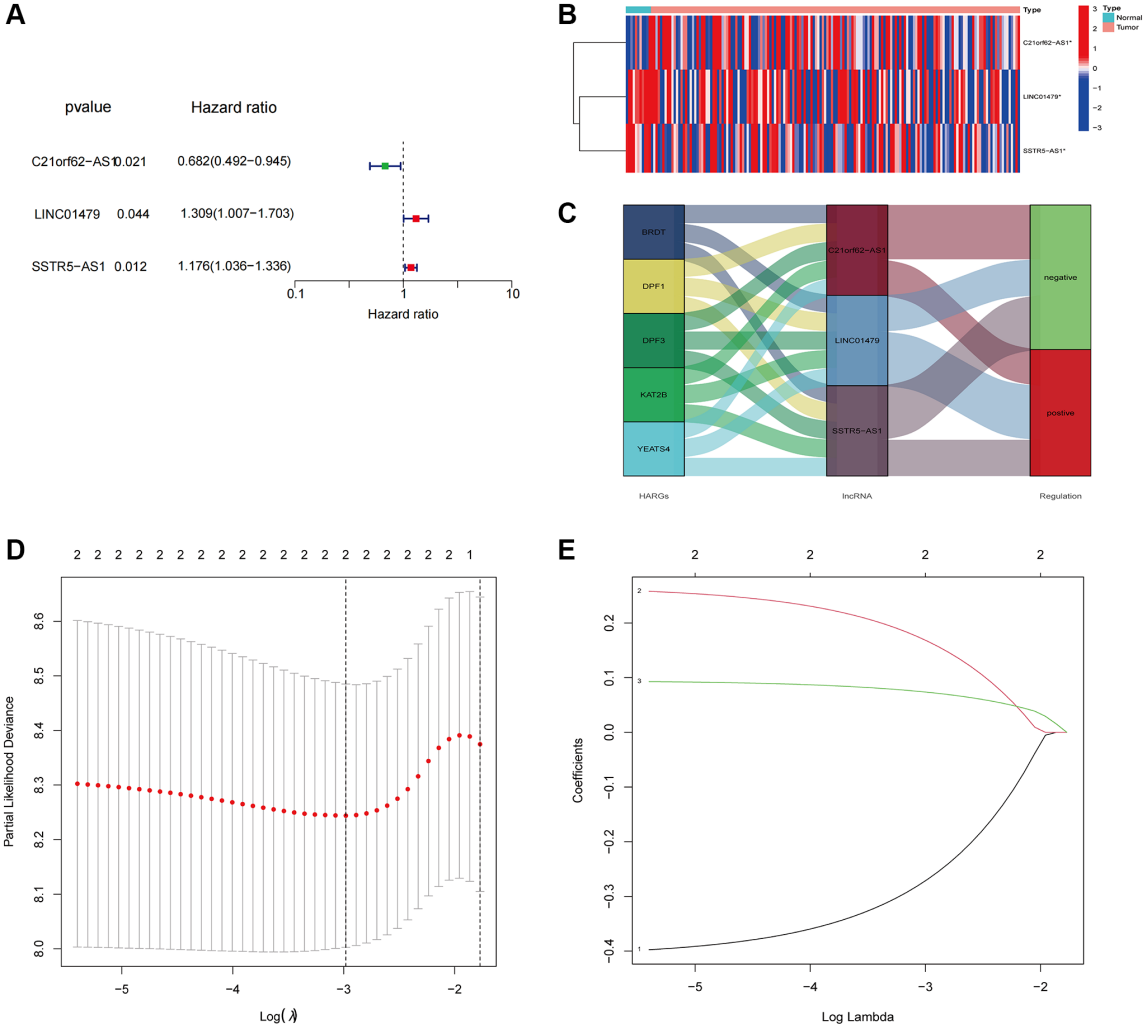
**Constructed a HARlncRNA risk signature in the TCGA cohort.** (**A**) Forest plot of 3 prognostic-related HARlncRNAs through univariate Cox analysis. (**B**) Expression patterns of 3 prognostic HARlncRNAs in normal and tumor tissues. (**C**) The Sankey diagram displayed the relationship between the 5 HARGs, 3 HARlncRNAs and ESCA prognosis. (**D**) Tuning parameter (λ) selection in LASSO model using cross-validation. (**E**) The LASSO coefficient profile of prognostic HARlncRNAs.

We conducted univariate Cox regression analysis on 7 HARlncRNAs and identified 3 HARlncRNAs that are linked to ESCA prognosis (Figure 3A). Figure 3B displays a heatmap of the 3 selected prognostic HARlncRNAs. Figure 3C depicts a mulberry plot that illustrates the association between 5 HARGs, 3 HARlncRNAs, and ESCA prognosis. Subsequently, we built a risk signature model for ESCA using LASSO regression analysis (Figure 3D, 3E). The results revealed that 2 HARlncRNAs were employed in constructing this risk signature. Ultimately, the risk score was computed by combining the expression values of the 2 HARlncRNAs with the risk regression coefficient, as follows: risk score = (−0.32098 × *C21orf62-AS1*) + (0.14225 × *SSTR5.AS1*).

### Validation of signature based on two HARlncRNAs

**Figure 4:**
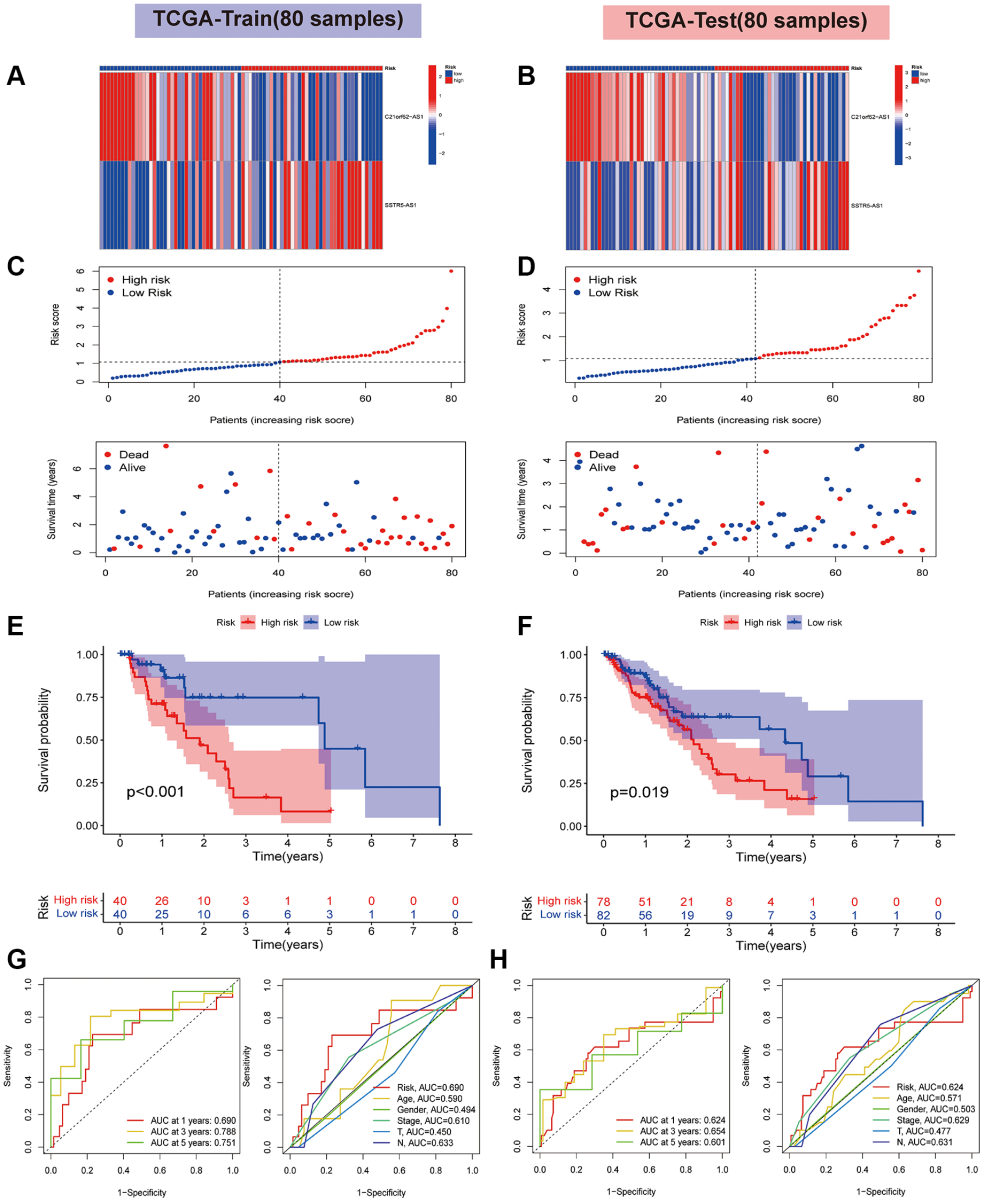
**Validation of risk signature based on two HARlncRNAs in the TCGA cohort.** (**A**) The heatmaps of prognostic 2 genes signature in the training set. (**B**) The heatmaps of prognostic 2 genes signature in the test set. (**C**) Risk score distribution plot showed the distribution of high-risk and low-risk LUAD patients in the training set. Scatter plot showed the correlation between the survival status and risk score of LUAD patients in the training set. (**D**) Risk score distribution plot showed the distribution of high-risk and low-risk LUAD patients in the test set. Scatter plot showed the correlation between the survival status and risk score of LUAD patients in the test set. The survival analysis in the training set (**E**) and test set (**F**). ROC curve analysis of the accuracy of the model to predict patient prognosis at 1, 3, and 5 years in the training (**G**) and test sets (**H**).

All samples from the TCGA-ESCA cohort were randomly allocated into a training set and a test set using the outcomes of LASSO regression analysis. Using the median risk score as a basis, the two datasets were further categorized into two risk subgroups: a high-risk group and a low-risk group. As depicted in Figure 4A, 4B, the heatmap illustrates the differential expression of the two HARlncRNAs in the two subgroups. Figure 4C, 4D display the risk score and survival status for each ESCA patient. Kaplan-Meier analysis revealed that patients in the high-risk group had shorter OS and median survival compared to those in the low-risk group (Figure 4E, 4F). As indicated in Figure 4G, the area under the curve (AUC) values for ROC analysis at 1 year, 3 years, and 5 years were 0.690, 0.788, and 0.751, respectively, in the training set. Additionally, in the test set, the risk signature demonstrated a favorable prognostic value (Figure 4H). All the aforementioned findings indicate that the high-risk group had an unfavorable prognosis, implying that the risk score of the model we constructed may have a crucial role in ESCA progression. To assess the applicability of the constructed risk model derived from the TCGA-ESCA cohort, we introduced an independent GEO dataset (GSE53624) as an external validation set. The model also demonstrated high sensitivity and effectiveness in the validation set (Supplementary Figure 1). In summary, these findings indicate that the risk model can predict overall survival relatively accurately.

**Figure 5:**
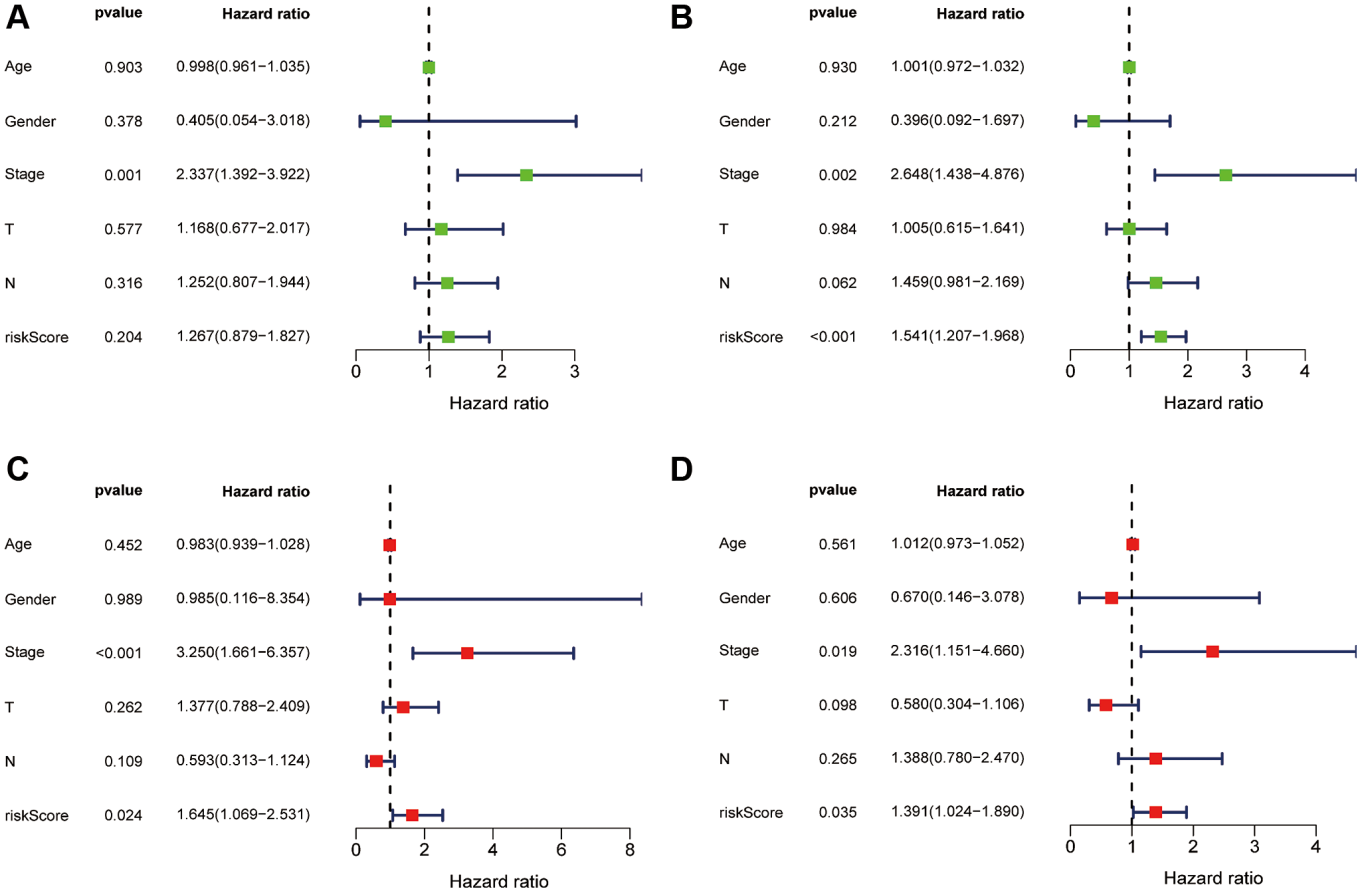
**Independent prognostic value of risk score.** Univariate Cox regression analysis of risk score in the training (**A**) and test sets (**B**). Multivariate Cox regression analysis also confirmed risk score as an independent prognostic factor for the training (**C**) and test sets (**D**).

Subsequently, we conducted univariate (Figure 5A, 5B) and multivariate Cox regression analyses (Figure 5C, 5D), which confirmed that the risk score and stage were independent prognostic factors (*P* < 0.05). In summary, our findings suggest that the prognostic risk score signature can be used independently and with confidence to predict survival outcomes in ESCA patients.

### Development of a clinical nomogram

**Figure 6:**
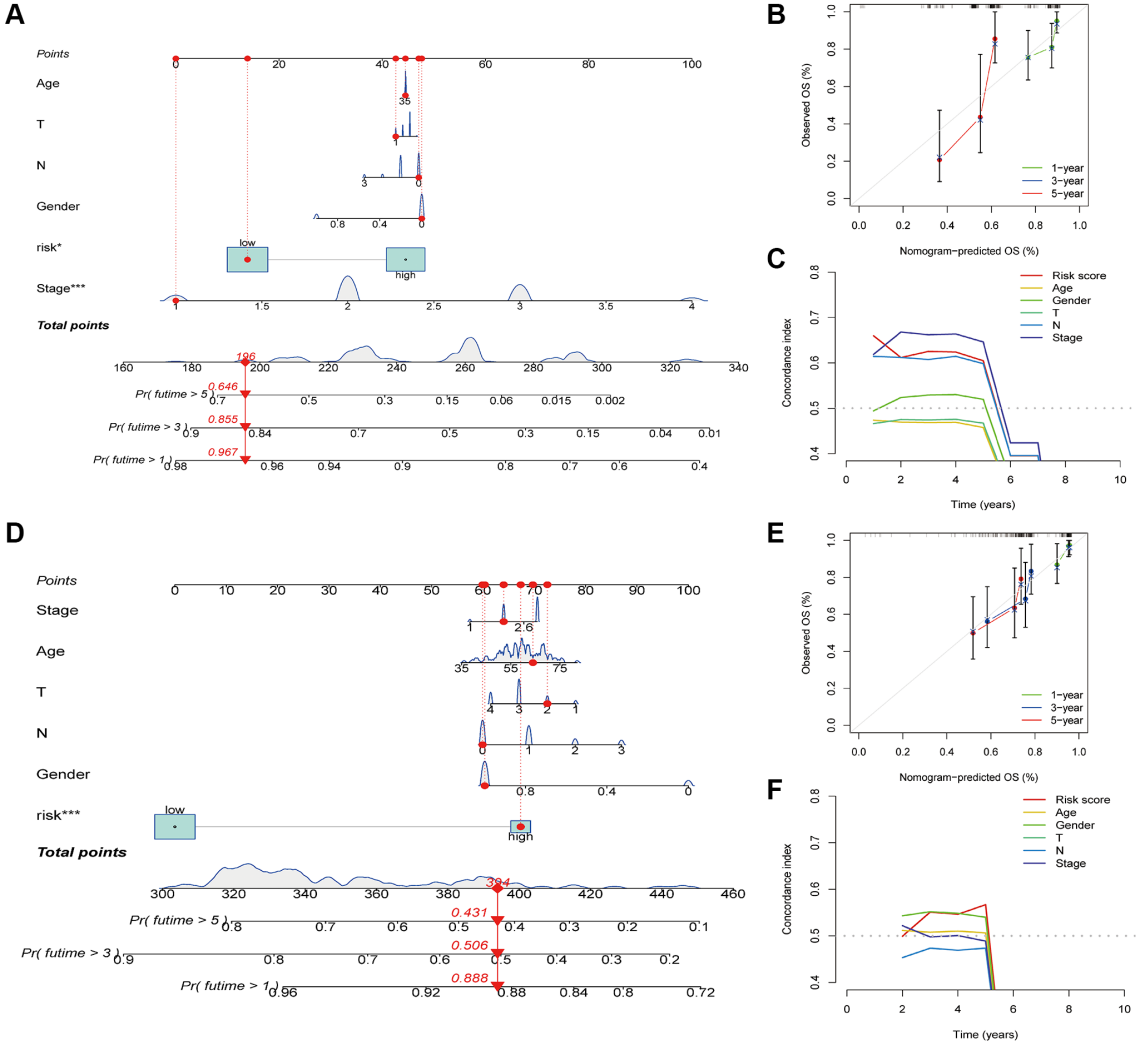
**Construction and verification of nomogram.** (**A**) Nomogram construction based on the risk score and ESCA-related clinical parameters in the TCGA-ESCA cohort. (**B**) Calibration plots of the nomogram in the TCGA-ESCA cohort. (**C**) C-index curves of the nomogram in the TCGA-ESCA cohort. (**D**) Nomogram construction based on the risk score and ESCA-related clinical parameters in the GSE53624 cohort. (**E**) Calibration plots of the nomogram in the GSE53624 cohort. (**F**) C-index curves of the nomogram in the GSE53624 cohort. ^*^P < 0.05; ^**^P < 0.01; ^***^P < 0.001.

Subsequently, we created nomograms to predict OS using clinical parameters and the risk score in the TCGA-ESCA (Figure 6A) and GSE53624 (Figure 6D) cohorts. The calibration plot, used for internally validating the nomogram, demonstrated a strong agreement between the predicted probabilities from the nomogram and the actual observations of 1-, 3-, and 5-year OS (data for the TCGA-ESCA and GSE53624 cohorts are depicted in Figure 6B, 6E, respectively). The C-index curves further confirmed the nomogram’s high accuracy in predicting survival probabilities (Figure 6C, 6F).

### Functional enrichment analysis of HARlncRNA target genes

**Figure 7:**
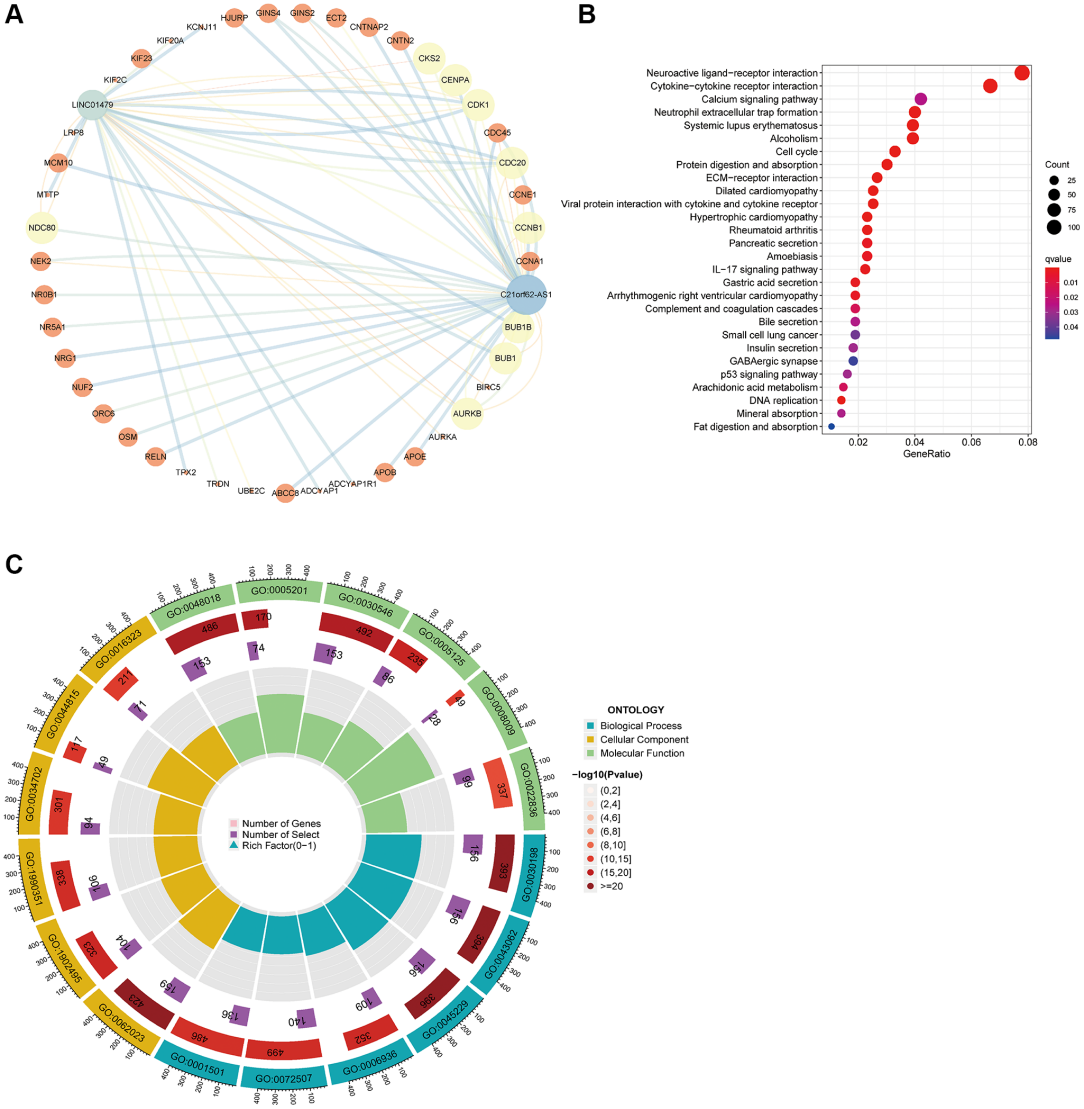
**Functional enrichment analysis of lncRNA target genes.** (**A**) Regulatory network of two lncRNA and core target genes. (**B**) KEGG enrichment analysis of core target genes. (**C**) GO enrichment analysis results of 74 core target genes.

Initial target gene prediction was carried out using StarBase, lncLocator, and LncRNA2Target for the two HARlncRNAs associated with risk. This was followed by the construction of a lncRNA-core target gene regulatory network using Cytoscape (Figure 7A), and subsequent enrichment analysis of the genes within the regulatory network. KEGG enrichment analysis results revealed that the core target genes of the two HARlncRNAs were primarily associated with pathways such as Systemic lupus erythematosus, Neuroactive ligand-receptor interaction, Cytokine-cytokine receptor interaction, and others (Figure 7B). The GO analysis indicated that the 74 core target genes primarily pertained to biological functions related to skeletal system development, transporter complexes, receptor ligand activity, and more (Figure 7C).

### Immunity analysis

**Figure 8:**
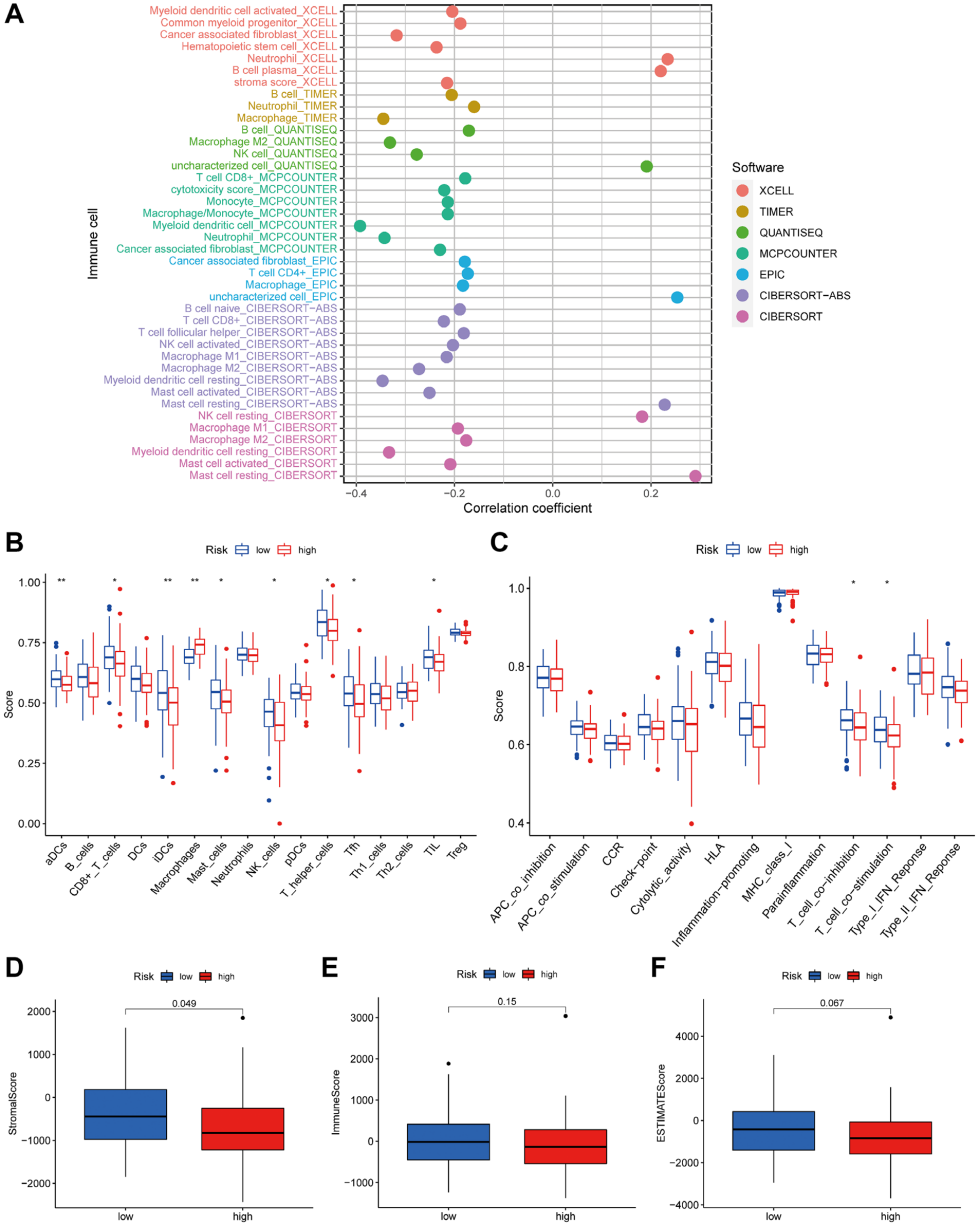
**The low- and high-risk groups display different immune statuses.** (**A**) The correlation of tumor-infiltrating cells with risk score using 7 algorithms. Immune cell infiltration (**B**) and immune-related functions or pathways (**C**) between the high- and low-risk groups. The stromal (**D**), immune (**E**) and ESTIMATE scores (**F**) between the high- and low-risk groups. ^*^P < 0.05; ^**^P < 0.01.

As stromal and immune cells in the TME significantly influence tumor progression, treatment effectiveness, and clinical outcomes, we compared TME differences between high-risk and low-risk groups. To gain a deeper insight into the TME, we assessed the extent of infiltration by 22 types of immune cells using seven distinct algorithms (TIMER, CIBERSORT, CIBERSORT-ABS, QUANTISEQ, MCP-counter, XCELL, and EPIC). As expected, the presence of cytotoxic immune cells, including CD4+T and CD8+T cells, declined as the risk score increased (Figure 8A). According to the ssGSEA algorithm, the low-risk group exhibited greater immune cell infiltration and a richer set of immune-related functions and pathways compared to the high-risk group (Figure 8B, 8C). The ESTIMATE algorithm analyzed the aforementioned results and determined that the low-risk group had higher immune scores, stromal scores, and overall ESTIMATE estimated scores (Figure 8D–8F).

### Association with TMB

**Figure 9:**
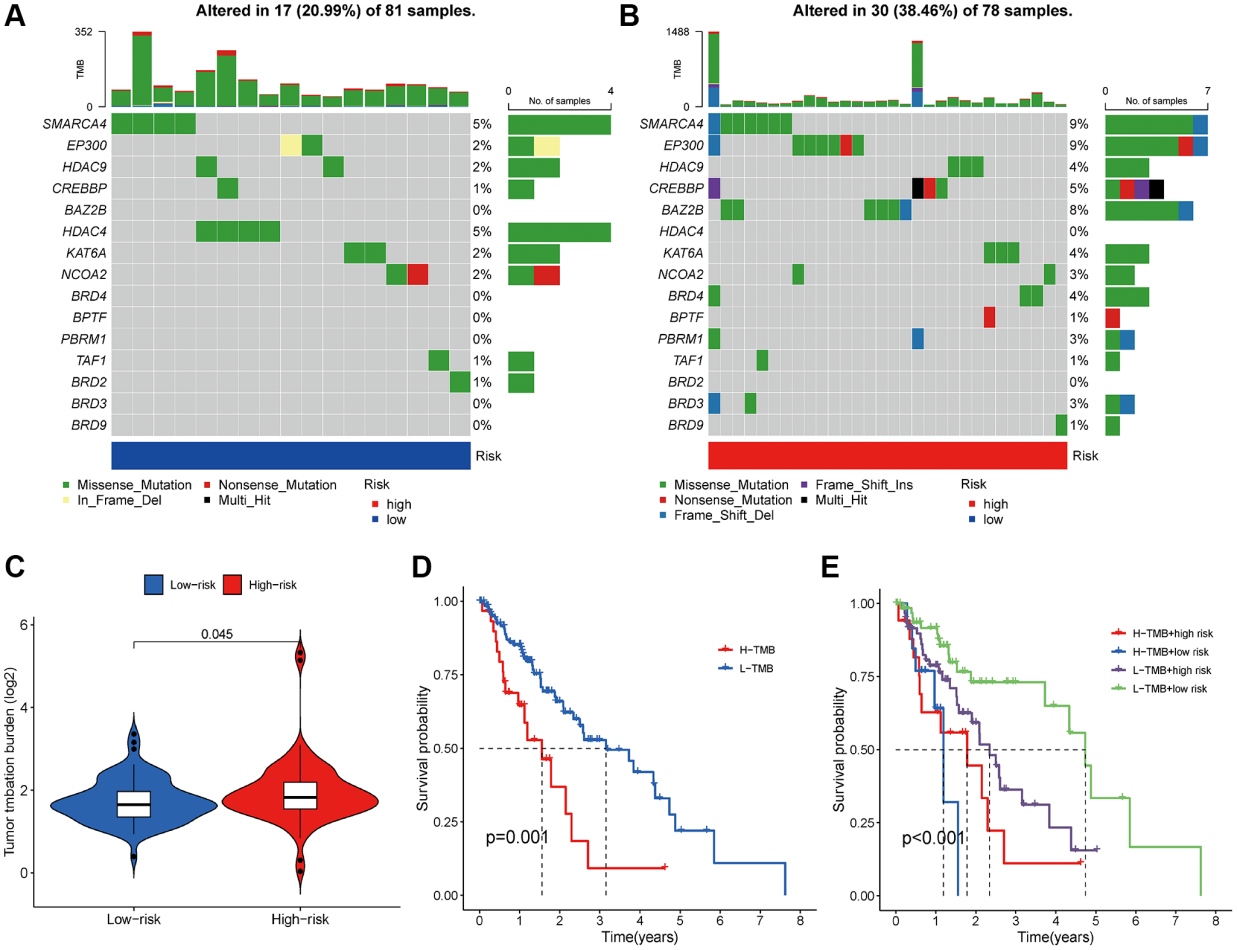
**Analysis of TMB between different risk groups.** (**A**, **B**) Top 10 mutated genes in different risk subgroups. (**C**) The differences of TMB in low- and high-risk groups. (**D**) Kaplan-Meier survival analysis of TMB. (**E**) Effects of the risk score combined with TMB on the overall survival.

In the TCGA dataset, we computed the TMB for all samples. It was observed that the TMB was higher in the high-risk group (Figure 9C). Subsequently, we used waterfall plots to visualize mutations in the high-risk and low-risk groups separately. The results revealed that the top 10 mutated genes were *SMARCA4*, *EP300*, *HDAC9*, *CREBBP*, *BAZ2B*, *HDAC4*, *KAT6A*, *NCOA1*, *BRD4*, and *BPTF* in both groups (Figure 9A, 9B). Missense mutations were the most prevalent somatic mutational type. The mutation frequency was markedly higher in the high-risk group (38.46%) compared to the low-risk group (20.99%). Subsequently, we conducted a Kaplan-Meier analysis to evaluate the impact of combining risk score and TMB on survival. The results indicated that the high-TMB group had a shorter overall survival than the low-TMB group (Figure 9D). More importantly, patients with both a low risk score and low TMB had significantly longer OS than those with high risk scores and high TMB (Figure 9E).

### Estimation of the HARlncRNA signature in immunotherapy response

**Figure 10:**
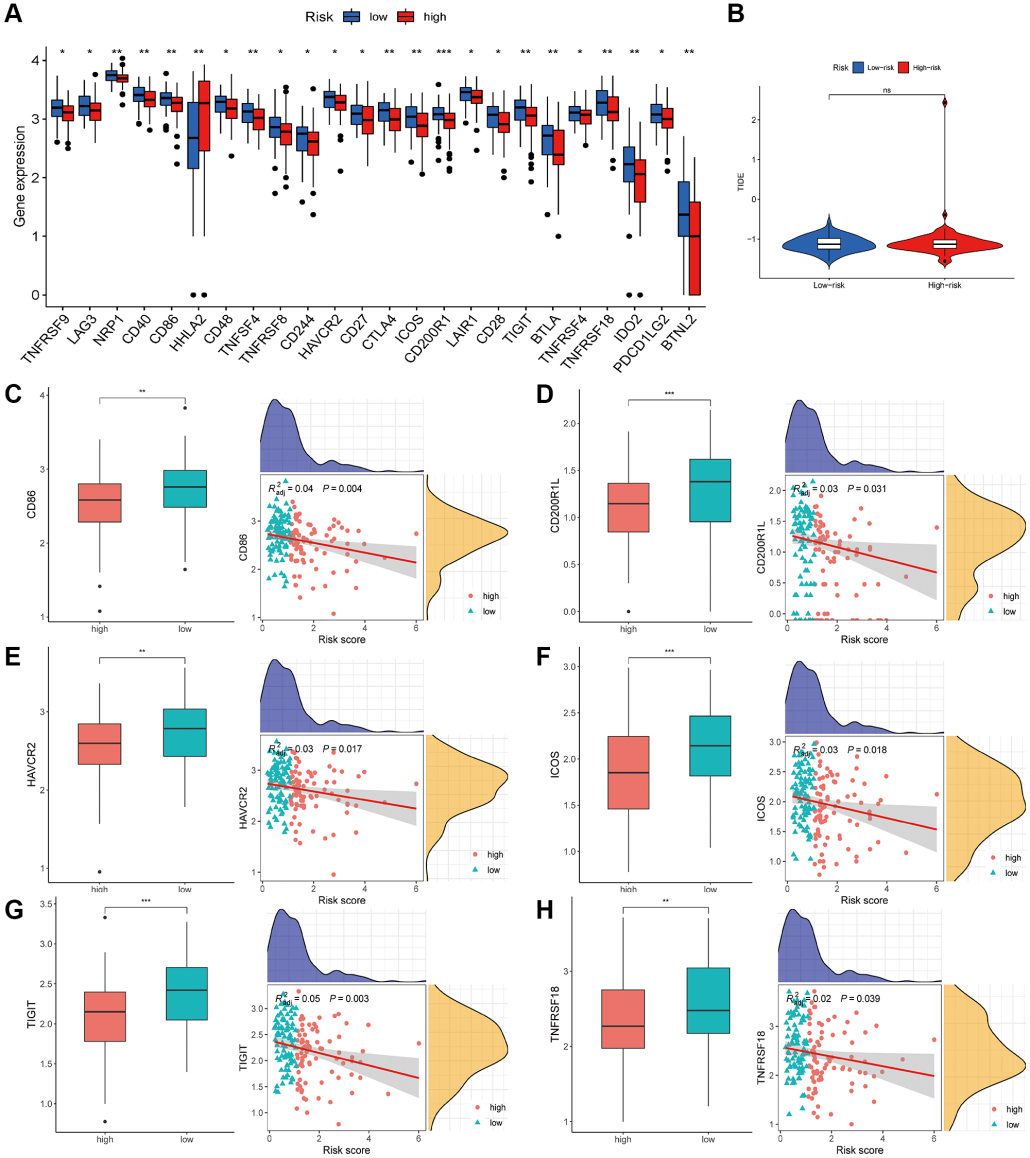
**Estimation of the HARlncRNA signature in immunotherapy response.** (**A**) Expression of ICGs in different risk groups. (**B**) Differences of TIDE score between the high- and low-risk groups. (**C**–**H**) Correlation of risk score with immune checkpoints. ^*^P < 0.05; ^**^P < 0.01; ^***^P < 0.001.

ICGs play a crucial role in regulating immune homeostasis and autoimmunity. To explore the relationship between risk scores and ICGs, we compared the expression levels of 24 ICGs in the two risk subgroups. All genes, except *HHLA2*, were significantly upregulated in the low-risk group compared to the high-risk group (Figure 10A). The Tumor Immune Dysfunction and Exclusion (TIDE) score is a novel computational framework developed by Peng Jiang and colleagues. It is designed to comprehensively evaluate mechanisms of tumor immune escape and is considered an effective alternative to single biomarkers for predicting the therapeutic effectiveness of immune checkpoint inhibitors. We also applied the TIDE scoring mechanism in our study, but we found no significant difference in TIDE scores between the two risk subgroups (Figure 10B). Lastly, we conducted additional investigations into the relationship between ICGs and the risk score. The results revealed a negative correlation between the risk score and the expression of several ICGs, including *CD86*, *CD200R1L*, *HAVCR2*, *ICOS*, *TIGIT*, and *TNFRSF18* (Figure 10C–10H).

### Drug sensitivity analysis

**Figure 11:**
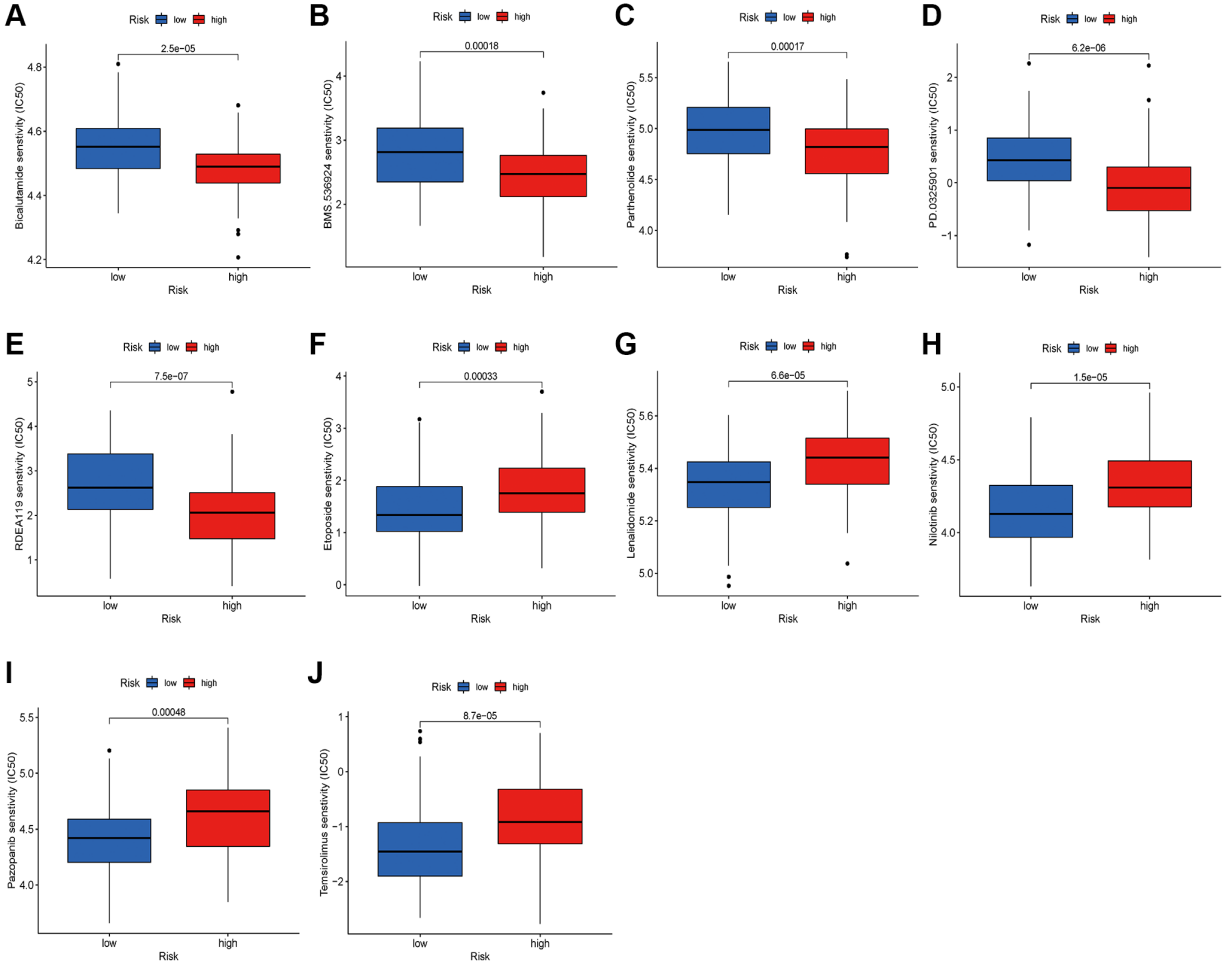
**Relationships between risk score and therapeutic sensitivity.** (**A**) Bicalutamide. (**B**) BMS.536924. (**C**) Parthenolide. (**D**) PD.0325901. (**E**) RDEA119. (**F**) Etoposide. (**G**) Lenalidomide. (**H**) Nilotinib. (**I**) Pazopanib. (**J**) Temsirolimus.

Patients with inoperable ESCA may benefit from chemotherapy and targeted therapy, which can curb tumor advancement and enhance their prognosis. Additionally, it is frequently employed as postoperative adjuvant therapy to prevent tumor recurrence and metastasis. The sensitivity of tumor cells to medications is a pivotal determinant of drug effectiveness. To assess the risk score’s utility as a biomarker for predicting treatment responses in ESCA patients, we analyzed IC50 values for over 200 anticancer drugs, utilizing data from the CGP database in conjunction with the risk score. Our findings suggest that patients with high-risk scores may exhibit a favorable response to BMS.536924, RDEA119, Bicalutamide, Parthenolide, and PD.0325901, whereas those with low-risk scores may respond more positively to Etoposide, Lenalidomide, and several targeted therapy agents, such as Temsirolimus, Nilotinib, and Pazopanib (Figure 11A–11J).

### Validation of the expression levels of two-lncRNA signature in ESCA tissues

**Figure 12:**
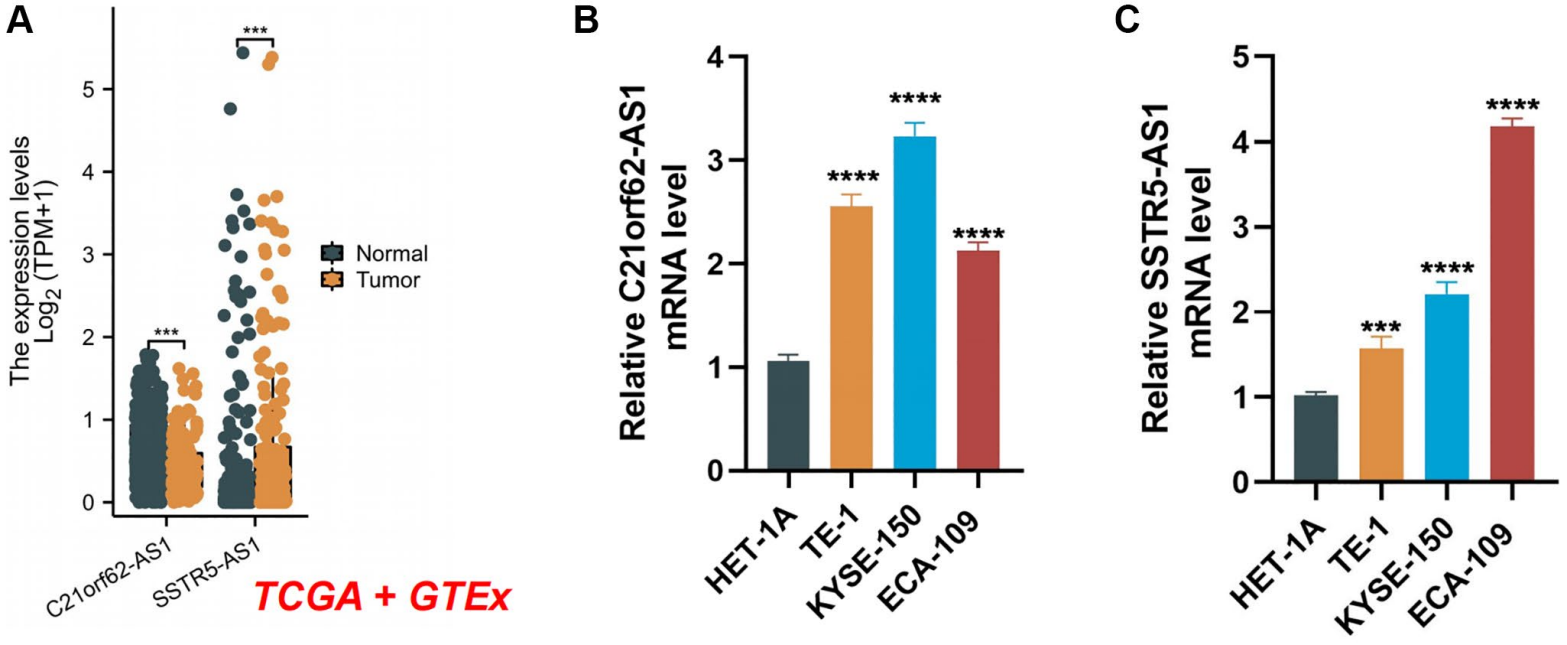
**The expression levels of two signature lncRNAs.** (**A**) The mRNA expression profile of two lncRNA in tumor tissues from the TCGA database and normal esophageal tissues from the TCGA and GTEx databases. (**B**, **C**) Further verification of the expression levels of two signature lncRNAs in human ESCA cancer cell lines and human normal esophageal epithelial cell line by qRT-PCR analysis. ^***^P < 0.001; ^****^P < 0.0001.

We examined the clinical significance of the two lncRNAs in the model by validating their mRNA expression levels using data from the TCGA and GTEx databases. As depicted in Figure 12A, the expression levels of *C21orf62-AS1* and *SSTR5.AS1* were significantly elevated in ESCA tissues. Furthermore, qRT-PCR results showed a significant increase in the expression levels of *C21orf62-AS1* and *SSTR5.AS1* in human ESCA cells compared to normal human esophageal epithelium cells (Figure 12B, 12C). In summary, these results provide additional confirmation of the stability and reliability of the risk signature.

## DISCUSSION

Recently, significant attention has focused on the epigenetic modifications of lncRNAs. Importantly, substantial evidence has highlighted the significance of epigenetic modifications in lncRNAs in the development and progression of cancer [24, 25]. Zhang et al. discovered that *ALKBH5* promotes invasion and metastasis in gastric cancer by reducing methylation of the lncRNA *NEAT1* [26]. Utilizing bioinformatics techniques, a prognostic model based on seven HARlncRNAs has demonstrated its ability to predict both the prognosis and immune response in lung adenocarcinoma [27]. The data collected thus far regarding lncRNA’s role in epigenetic regulation likely represents only the beginning of this emerging field. However, to our knowledge, no prior study has investigated the connections between HARlncRNAs and the evaluation of prognosis and the TME in ESCA.

In our current study, we analyzed DElncRNAs in 162 ESCA tissues and 11 normal tissues from the TCGA database, resulting in the identification of 653 DElncRNAs. Through Pearson correlation analysis, we identified a total of 7 lncRNAs that showed correlations with acetylation regulators. When combined with clinical prognosis data, we selected 3 lncRNAs that are related to prognosis in the TCGA dataset. Next, we developed a risk signature for ESCA using LASSO regression analysis and identified *C21orf62-AS1* and *SSTR5.AS1* as potential biomarkers for assessing the prognosis of ESCA patients. Chen et al. found that *C21orf62-AS1* is closely linked to predicting gastric cancer recurrence and plays a role in regulating various biological functions and signaling pathways related to gastric cancer. Suppression of *C21orf62-AS1* led to apoptosis in gastric cancer cells. Furthermore, elevated expression of *C21orf62-AS1* was associated with significantly reduced median survival time in gastric cancer patients [28]. Wang et al. [29] found that abnormal methylation-induced downregulation of lncRNA *SSTR5-AS1* promotes the progression and metastasis of laryngeal squamous cell carcinoma. Hu et al. [30] found that lncRNA *SSTR5-AS1* predicts a poor prognosis and contributes to ESCA progression. While many of its functions remain unknown, we have shown its connection to cancer pathology and malignant progression through extensive data analysis.

Following the construction of the risk model, we assessed the risk score for each tumor sample based on the expression of HARlncRNAs in the risk signature. Based on the risk score, we categorized the samples into high-risk and low-risk groups. We identified the risk score as an independent risk factor and validated it across the training, test, and validation sets. This suggests that histone acetylation modification is a reliable method for comprehensively assessing ESCA.

Given the strong association between histone acetylation patterns, immunotherapy response, and the TME, we investigated the potential therapeutic impact of the HARlncRNA risk signature. Our analysis uncovered that the low-risk group exhibited higher levels of immune cell infiltration and increased expression of immunomodulators, including classical immune checkpoint molecules. Patients in the low-risk group showed significantly higher StromalScore, ImmuneScore, and ESTIMATEScore levels than those in the high-risk group. This implies that patients in the low-risk group might have a more favorable response to immunotherapy. Given the potential link between histone acetylation modification and immune regulation, we delved deeper into the correlation between acetylation-related scores and TMB. TMB has gained acceptance as a predictor for immunotherapy in advanced NSCLC and has been endorsed by the latest NCCN guidelines [31]. Our results demonstrate a significant correlation between the acetylation-related score and TMB. Patients with high TMB and acetylation-related scores experience worse prognoses, revealing an underlying and indirect connection between acetylation modification and immunotherapy in ESCA. These findings imply that our model can effectively predict the outcomes of immunotherapy in ESCA patients.

To our knowledge, this study presents the initial landscape of HARlncRNAs in ESCA and delves into the diagnostic and biological functions of the identified biomarkers. However, our study does possess certain inevitable limitations. Firstly, it’s important to recognize that the HARlncRNA risk signature was retrospectively developed from publicly accessible databases, possibly leading to inherent selection bias. To determine the applicability and strength of our results, conducting thorough prospective and multicenter clinical studies is essential. Additionally, it’s vital to recognize the absence of key clinical factors like chemoradiotherapy and surgery in the analyzed datasets, highlighting the need to include them in future research. This limitation could have affected the accuracy of treatment response and disulfidptosis state analyses. Moreover, conducting additional *in vivo* and *in vitro* experiments is essential for a comprehensive understanding of the roles of signature genes in the context of the disease.

## CONCLUSION

In our study, we developed a HARlncRNA risk signature that holds significant predictive value for prognosis and offers insights into the tumor microenvironment in ESCA.

## MATERIALS AND METHODS

### Data collection

We obtained RNA transcriptome sequencing data, somatic mutation data, copy number variation (CNV) data, and associated clinical information for ESCA from the TCGA database. In this study, we divided the TCGA-ESCA cohort into training and test sets. For result validation on the training set, we additionally retrieved an independent ESCA dataset (GEO: GSE53624) from the GEO database to serve as an external validation set. Read count values from each database were downloaded in the form of fragments per kilobase million (FPKM). To mitigate batch effects, we utilized the “Combat” function within the “SVA” R package. We retrieved literature on histone acetylation modification and curated 52 recognized HARGs, which are listed in Supplementary Table 1. We employed the “biomaRt” package in R to obtain the chromosomal location information of HARGs. We utilized the “TBtools” package to create a chromosomal distribution map of HARGs. We obtained CNV profiles of ESCA from the TCGA database, intersected these profiles with the selected HARGs, and used the R package “ggplot2” for data visualization.

### Differential expression analysis of HARGs and acquisition of HARlncRNAs

To discover HARGs and lncRNAs contributing to ESCA progression, we conducted differential expression analysis comparing 11 normal tissues with 162 tumor tissues in the TCGA-ESCA cohort. Genes and lncRNAs with |log2 Fold Change (FC) | > 1 and *P* < 0.05 were defined as differentially expressed. Furthermore, we intersected the known 52 HARGs with the 5 genes identified from the differentially expressed genes. Then, lncRNAs showing a significant correlation with these 5 HARGs were screened using the Pearson correlation analysis algorithm. In the end, we identified 7 HARlncRNAs for subsequent bioinformatics analysis.

### Construction and validation of the prognostic signature

We conducted univariate Cox regression analysis on the differentially expressed HARlncRNAs to assess their prognostic significance. To prevent overfitting, we also carried out LASSO Cox regression with 10,000 iterations, employing the “glmnet” package. The lncRNAs identified through LASSO regression were utilized for risk score calculation. The risk score was computed using the following formula: risk score = (expression level of gene1 × coefficient of gene1) + (expression level of gene2 × coefficient of gene2) + ... + (expression level of gene n × coefficient of gene n). ESCA patients were stratified into two subgroups based on the median risk score, which comprised the high-risk and low-risk groups. Survival curves were generated through Kaplan-Meier analysis and the log-rank test, utilizing the “survival” R package to evaluate prediction accuracy. Receiver operating characteristic (ROC) curves for the risk scores were constructed with the “timeROC” R package.

### Nomogram construction and validation

To address the clinical utility of the histone acetylation-related score, a nomogram was developed using a Cox regression model. The nomogram incorporated age, gender, grade, stage, and the histone acetylation-related score. The model’s performance was assessed using calibration and the concordance index (C-index). To assess the alignment between observed and estimated survival probabilities, bias-corrected calibration for 3 and 5-year overall survival rates was conducted using 1,000 bootstrap resamples, employing the “rms” package. Calibration was determined using the “calibrate” function with the parameter settings “cmethod = KM, method = boot, m = 80”. Model discrimination was assessed using Harrell’s C-index, where a higher C-index value indicated superior model-fitting performance.

### Functional enrichment analysis

We used the default parameters of three databases: StarBase (https://rnasysu.com/encori/), LncRNA2Target (http://bio-annotation.cn/lncrna2target/search.jsp), and lncLocator (http://www.csbio.sjtu.edu.cn/bioinf/lncLocator/) to simultaneously predict target genes for HARlncRNAs. We then combined the resulting predictions to determine the target genes of HARlncRNAs. To gain a deeper understanding of the function of HARlncRNA target genes and the predominant enriched signaling pathways, we conducted enrichment analysis using the R package “clusterProfiler”. GO enrichment results were graphically represented with the R package “circlize”, while KEGG enrichment results were depicted using the R package “ClusterProfiler”.

### Immune landscape analysis

For immune cell analysis, we employed multiple algorithms, including TIMER, CIBERSORT, CIBERSORT-ABS, QUANTISEQ, MCP-counter, XCELL, and EPIC, to assess the levels of immune cell infiltration in various samples. We applied the ESTIMATE algorithm to compute stromal score, ESTIMATE score, and immune score, which provide insights into the tumor microenvironment. We determined the activity of immune cells, immune functions, and immune pathways for each sample through single-sample gene set enrichment analysis (ssGSEA).

### Correlations of histone acetylation-related score with tumor mutational burden (TMB), immune checkpoint genes (ICGs), and immunotherapy response

Patient response rates to immunotherapy have been associated with both TMB and ICGs. We computed the TMB for each ESCA sample using somatic mutation data processed with VarScan2 software in the TCGA-ESCA cohort. Next, we compared TMB differences between the high-risk and low-risk groups. We visualized somatic mutations in both risk groups using the “maftools” R package. Additionally, we investigated the influence of combining the risk score with TMB on the survival of ESCA patients. The expression levels of ICGs may have associations with the responses to treatment with immune checkpoint inhibitors. We assessed the disparities in gene expression levels between the high-risk and low-risk groups to investigate the link between the risk score and the response to immune checkpoint inhibitors. To assess the effectiveness of immunotherapy for ESCA patients, we utilized the tumor immune dysfunction and exclusion (TIDE) algorithm, available at http://tide.dfci.harvard.edu.

### Drug sensitivity analysis

The “pRRophetic” package is an algorithm developed using data from the Cancer Genome Project (CGP) database, containing information about how more than 700 cell lines respond to 138 different drugs. This algorithm is designed to predict how drugs will respond. We conducted drug sensitivity prediction by utilizing the internal algorithm within the “pRRophetic” package and applying the linearRidge method for ridge regression analysis. Additionally, by incorporating sample grouping data, we computed the half-maximal inhibitory concentration (IC50) values for each sample in response to different drugs. This facilitated the identification of drugs with varying sensitivities across different groups, offering valuable insights for future research.

### Cell culture and qRT-PCR analysis

We obtained the ESCA cell lines (TE-1, KYSE-150, and ECA-109), along with the human normal esophageal epithelial cell line (HET-1A), from the Cell Repository of the Chinese Academy of Sciences in Shanghai, China. All cell lines were cultured in RPMI-1640 medium supplemented with 10% Fetal Bovine Serum (FBS), streptomycin (100 U/mL), and penicillin (100 U/mL) at 37°C in a 5% CO_2_ environment.

We isolated total RNA from cell lines using 1 mL of TRIzol^®^, and then we synthesized complementary DNA (cDNA) with reverse transcriptase derived from avian medulloblastoma virus and random primers, following TAKARA’s instructions. We conducted qRT-PCR using SYBR Premix Ex Taq II from Takara in Shiga, Japan. Data analysis was carried out based on 2^−ΔΔCT^ values.

### Statistical analysis

We performed all statistical analyses using R software (v.4.0.0). The preceding section provided detailed statistical methods for processing transcriptome data. A *p*-value below 0.05 was considered statistically significant.

## Supplementary Materials

Supplementary Figure 1

Supplementary Table 1
