# Supplementary material for: Novel histone acetylation-related lncRNA signature for predicting prognosis and tumor microenvironment in esophageal carcinoma
Source: Aging (Albany NY). 2024 Mar 13;16(6):5163–83. doi: 10.18632/aging.205636 (PMC11006502; doi:10.18632/aging.205636)
Supplement: Supplementary Figure 1 [file aging-16-205636-s001.pdf]

SUPPLEMENTARY FIGURE

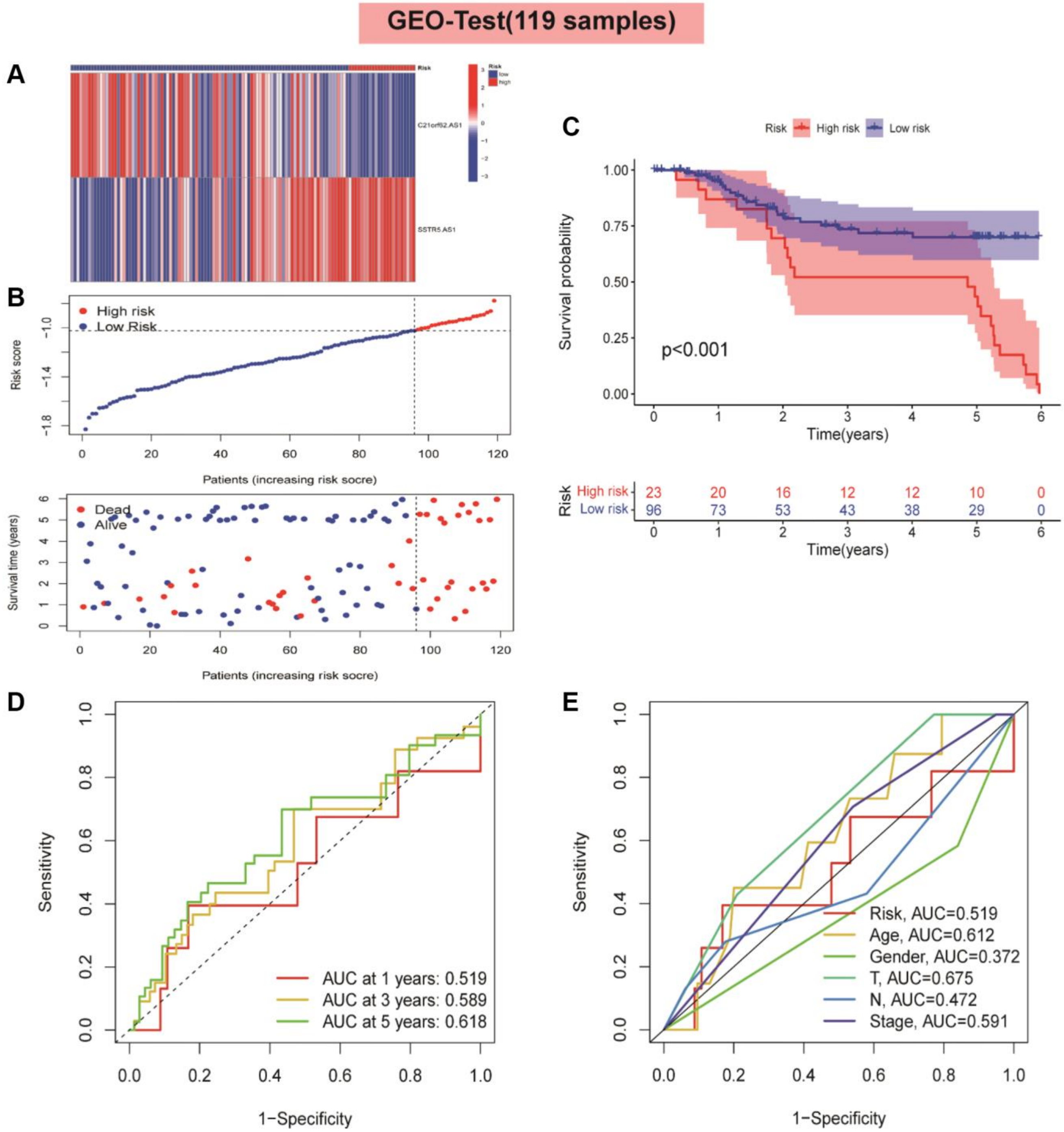

Supplementary Figure 1. Validation of risk signature based on two HARlncRNAs in the GEO cohort. (**A**, **B**) The risk score distribution, survival status, and heatmap for the expressions of the two lncRNAs in the validation set. (**C**) The survival analysis in the validation set. (**D**, **E**) ROC curve analysis of the accuracy of the model to predict patient prognosis at 1, 3, and 5 years in the validation sets.
