# Supplementary material for: Novel histone acetylation-related lncRNA signature for predicting prognosis and tumor microenvironment in esophageal carcinoma
Source: Aging (Albany NY). 2024 Mar 13;16(6):5163–83. doi: 10.18632/aging.205636 (PMC11006502; doi:10.18632/aging.205636)
Supplement: Supplementary Table 1 [file aging-16-205636-s002.pdf]

SUPPLEMENTARY TABLE

Supplementary Table 1. Summary of 52 recognized histone acetylation-related genes.

Gene	Type
HDAC4	Histone acetylation
HDAC5	Histone acetylation
HDAC2	Histone acetylation
HDAC3	Histone acetylation
HDAC10	Histone acetylation
HDAC1	Histone acetylation
HDAC11	Histone acetylation
HDAC8	Histone acetylation
HDAC9	Histone acetylation
HDAC6	Histone acetylation
HDAC7	Histone acetylation
KAT8	Histone acetylation
EP300	Histone acetylation
KAT7	Histone acetylation
BRD4	Histone acetylation
BRD3	Histone acetylation
BRD2	Histone acetylation
CREBBP	Histone acetylation
SIRT2	Histone acetylation
KAT2B	Histone acetylation
KAT2A	Histone acetylation
KAT6B	Histone acetylation
KAT6A	Histone acetylation
HAT1	Histone acetylation
BRDT	Histone acetylation
TAF1	Histone acetylation
YEATS4	Histone acetylation
BAZ2B	Histone acetylation
ATAD2B	Histone acetylation
DPF1	Histone acetylation
DPF2	Histone acetylation
DPF3	Histone acetylation
BPTF	Histone acetylation
PBRM1	Histone acetylation
SMARCA2	Histone acetylation
SMARCA4	Histone acetylation
ATF2	Histone acetylation
CIITA	Histone acetylation
KAT5	Histone acetylation
BRD9	Histone acetylation
BRD7	Histone acetylation
NCOA1	Histone acetylation
NCOA2	Histone acetylation
NCOA3	Histone acetylation

SIRT4	Histone acetylation
OGA	Histone acetylation
SIRT5	Histone acetylation
SIRT6	Histone acetylation
SIRT7	Histone acetylation
SIRT1	Histone acetylation
SIRT3	Histone acetylation
CLOCK	Histone acetylation
